# Poison Plants

**Published:** 1879-08

**Authors:** 


### POISON PLANTS.

The most common of these in the United States are the poison ivy and poison sumac. *Treatment.*—A good dose of Rochelle salts, and keeping the surface moist with a solution of one drachm of acetate of lead in a pint of water; or a teaspoonful of baking soda in a pint of water, to be applied immediately after exposure before the symptoms develop. Extract of witch hazel has been highly recommended as a wash.

